# Thermostability as a highly dependent prion strain feature

**DOI:** 10.1038/s41598-019-47781-6

**Published:** 2019-08-06

**Authors:** Alba Marín-Moreno, Patricia Aguilar-Calvo, Mohammed Moudjou, Juan Carlos Espinosa, Vincent Béringue, Juan María Torres

**Affiliations:** 1 Centro de Investigación en Sanidad Animal (CISA-INIA), Valdeolmos, Madrid, Spain; 2 VIM, INRA, Université Paris-Saclay, 78350 Jouy-en-Josas, France; 3 Present Address: Department of Pathology, UC San Diego, La Jolla, CA 92093 USA

**Keywords:** Molecular biology, Prion diseases

## Abstract

Prion diseases are caused by the conversion of physiological PrP^C^ into the pathogenic misfolded protein PrP^Sc^, conferring new properties to PrP^Sc^ that vary upon prion strains. In this work, we analyze the thermostability of three prion strains (BSE, RML and 22L) that were heated at 98 °C for 2 hours. PrP^Sc^ resistance to proteinase K (PrP^res^), residual infectivity by mouse bioassay and *in vitro* templating activity by protein misfolding cyclic amplification (PMCA) were studied. Heated strains showed a huge loss of PrP^res^ and a radically different infectivity loss: RML was the most thermolabile strain (6 to 7 log10 infectivity loss), followed by 22L (5 log10) while BSE was the most thermostable strain with low or null infectivity reduction showing a clear dissociation between PrP^res^ and infectivity. These results indicate that thermostability is a strain-specific feature, measurable by PMCA and mouse bioassay, and a great tool to distinguish prion strains.

## Introduction

Prion diseases are fatal neurodegenerative diseases that affect numerous mammal species and include kuru, Creutzfeldt-Jakob disease (CJD), fatal familial insomnia (FFI) and Gerstmann-Sträussler-Scheinker disease (GSS) in humans, scrapie in sheep and goats, bovine spongiform encephalopathy (BSE) in cattle and chronic wasting disease (CWD) in cervids^1^. According to the Protein-Only Hypothesis, prion diseases are caused by the conversion of the physiological cellular prion protein (PrP^C^) into a pathogenic *β*-sheets enriched isoform (PrP^Sc^) that is able to self-propagate by recruiting and converting more PrP^C^ ^2^.

PrP^C^ conversion into PrP^Sc^ is a post-translational process where both isoforms share an identical amino acid sequence but differ in their conformation. This conformational change confers distinct physicochemical properties such as greater tendency to aggregate, greater insolubility in non-ionic detergents, partial resistance to protease digestion and high resistance to heat and chemical sterilization^3–7^. Notably, these properties vary upon prion agents which may result in distinctive prion disease phenotypes including incubation times, clinical signs, histopathological lesions and PrP^Sc^ deposition patterns in the brain. The existence of such prion strains has been proposed by many authors and are associated to the range of thermodynamically stable PrP^Sc^ conformers into which the PrP^C^ can misfold during prion conversion^8–10^. Many prion features, apart from the ones directly related to disease phenotype and mentioned above are given by the particular prion strain. In this way, the “stability” of prions, understood as the ability to support certain physical-chemical conditions retaining its specific strain features, could be considered as other differential trait between strains. Related to this concept of prion stability, a direct correlation between the fragility of yeast prion fibrils and their rate of replication have been reported^11–13^ and extended to mammalian prions from synthetic mammalian prions^14,15^. By contrast, ovine and human prions seem not to follow this rule^16,17^. In addition, conformational stability assay of 30 different prion isolates revealed a linear relationship between the concentrations of guanidine hydrochloride (Gdn-HCl) required to denaturing 50% of PrP^Sc^ molecules and their incubation times^15^. Prion stability has also been associated with the differential ability of prions to invade the central nervous system (CNS)^18^. This suggests that highly neuroinvasive prion strains may be conformationally unstable in denaturing conditions and efficiently form diffuse, non-fibrillar PrP aggregates in the CNS, which produces a rapid progression to terminal disease in mice. On the other hand, weakly neuroinvasive strains may form dense, fibrillar plaques and mice progress to terminal disease more slowly^18^.

In this work, we analyze the stability of three prion strains from the point of view of stability against heat treatment (thermostability). For that purpose, three different mouse-adapted prion strains (BSE, RML and 22L) were heated and/or proteinase K (PK) digested and the effect of these treatments on their biological and biochemical properties as well as on their infectivity and templating activity was studied. Our results suggest that heat treatment substantially reduced PK resistant PrP^Sc^ (PrP^res^) decreases prion infectious titer without changing any prion strain feature and that prion resistance to heat treatment is strain dependent, BSE being the most thermostable strain with low or null infectivity and templating activity decrease. Differential thermostability between strains combined with PMCA appears as a suitable method for strain typing.

## Results

### Differential effect of the heat treatment on the biochemical and biological properties of prion strains

**Figure 1:**
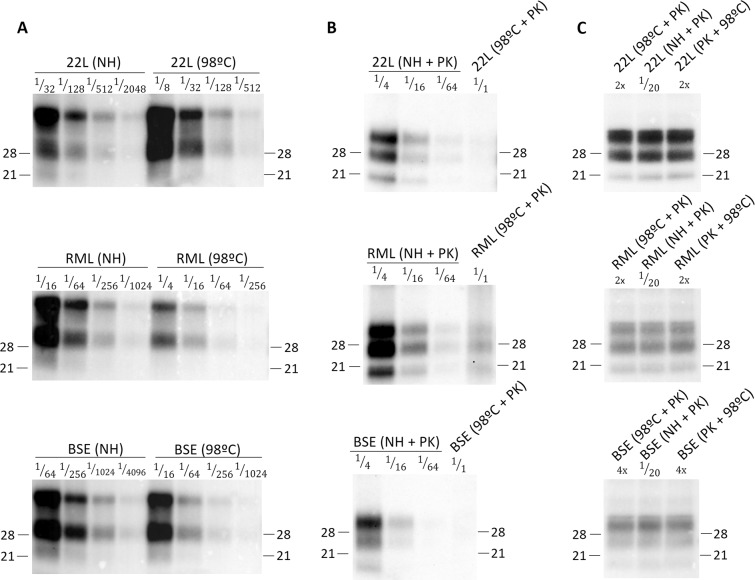
Total PrP and PrP^res^ levels of non-heated (NH) and heated (98 °C) and PK digested (PK) samples. (**A**) The relative amount of total PrP in non-heated (NH) and heated (98 °C) samples was quantified by serial dilutions in WB using the Sha31 mAb. (**B**) PrP^res^ quantification in non-heated (NH + PK) and heated (98 °C + PK) samples by serial dilutions in WB using the Sha31 mAb. Heated samples showed a general decrease on both total PrP and PrP^res^ levels. (**C**) Comparison of PrP^res^ glycoprofile between non-heated (NH + PK) and heated samples (98 °C + PK or PK + 98 °C) by loading equilibrated amounts of protein. To obtain comparable signals, it was necessary to load a 2x or 4x fold amount of heated samples against a 1/20 dilution of the unheated samples. The reduction in the amount of PrP^res^ in heated samples was independent of whether the PK treatment was done before (PK + 98 °C) or after heat treatment (98 °C + PK). In all cases, samples were digested using PK at a 40 µg/ml concentration. Serial dilutions were done by diluting the original sample into loading buffer. Molecular weights in kD are shown.

22L, RML and BSE inocula for this work were obtained by pooling brain homogenates of *Tga20* transgenic mice (10% weight/volume in PBS) after serial passage. *Tga20* transgenic mice were selected because its high overexpression of mouse PrP allow murine prion amplification with short mean survival times with a high sensitivity. These materials were subjected to heat treatment or heat treatment combined with PK digestion and then assayed for biochemical characteristics. First, Western blot (WB) analysis of the total PrP levels on the plain heated and non-heated samples were done (Fig. 1A). Heated samples showed a great reduction in their total PrP when compared to their non-heated counterparts. In the same line of results, WB analysis of the heated samples showed lower amounts of detectable PrP^res^ than their respective non-heated counterparts (at least 64 fold reduction for 22L and BSE while between 16 to 64 fold reduction was quantified for RML) (Fig. 1B). In addition, samples subjected first to PK treatment and then heat pulse were included in the study. Since heat treatment caused such a high reduction on the PK resistant population, we applied the heat pulse to a sole PK resistant PrP population to test if any differences with the previous samples can be found. These samples also showed the same amounts of detectable PrP^res^ than the plain heated ones (Fig. 1C). To obtain comparable signals, it was necessary to load a 2x or 4x fold amount of heated samples against a 1/20 dilution of the unheated samples. The reduction in the amount of PrP^res^ in heated samples was independent of whether the PK treatment was done before (PK + 98 °C) or after heat treatment (98 °C + PK). Thus, suggesting that the PrP termoresistant population is also PK resistant but only a very limited part of the PrP^res^ population is termoresistant.

These losses of both total PrP and PrP^res^ signals were in the same order of magnitude for all the strains which suggests that this degradation may not be strain-dependent. In addition, the biochemical signature of the samples treated with the different conditions showed no apparent difference in the WB PrP^res^ glycoprofile when compared to its non-heated controls (Fig. 1C).

Sha31 monoclonal antibody (mAb) was selected for the detection of PrP in WB because it binds the 144-WEDRYYRE-151 epitope of the mouse-PrP sequence^19^, which is in the core of the PrP^res^ and allows detection of the different PrP^res^ signatures with high sensitive. Other mAbs such as Saf84 (162-YRPVDQY-168 epitope of the mouse-PrP sequence)^19^ and 9A2 (98-WNK-100 epitope of the mouse-PrP sequence)^20^ were also tested producing very similar results (data not shown).

**Figure 2:**
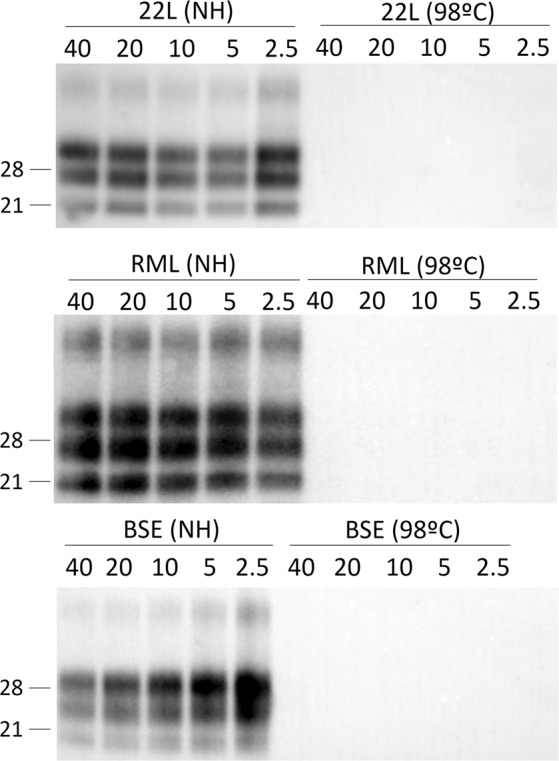
Analysis on PrP^res^ sensibility to PK treatment before and after heat treatment. PrP^res^ sensibility to PK treatment was analyzed by WB using the Sha31 mAb. Different concentration of PK ranging from 40 to 2.5 µg/ml were applied to non-heated and heated samples. Non-heated samples showed a high and stable resistance to PK while heated samples showed no PrP^res^ presence even at low concentrations of PK when only faint bands were detected. Molecular weights in kD are shown.

Finally, heated and non-heated samples were subjected to PK digestion using PK concentrations ranging from 2.5 to 40 µg/ml (Fig. 2). This will clarify if PrP^res^ reduction detected on heated samples by WB was due to direct degradation by heating or due to an increased sensitivity to PK as a consequence of a conformational change during the heating process. Sha31 antibody was selected for the PK assay because it binds the 144-WEDRYYRE-151 epitope of the mouse-PrP sequence^19^, which is in the core of the PrP^res^ and allows detection of several different PrP^res^ signatures with high sensitivity. As expected, non-heated isolates of all the three strains remained stable after PK digestion. No PrP^res^ signal was detected even when the heated samples were treated with low concentrations of PK.

We next studied whether heat treatment altered the strains biological properties. For that purpose, plain heated and unheated BSE, RML and 22L samples were intracerebrally inoculated in *Tga20* mice and mean survival time (ST) assessed. Mice inoculated with heated RML or heated 22L isolates showed 89% and 55% increased ST, respectively, compared to the animals inoculated with their correspondent original unheated samples (Table 1). By contrast, the heat treatment increased the ST of heated BSE inoculated mice by just a 7% compared to the animals inoculated with original unheated BSE, which is not statistically significant (Table 1).

**Table 1:** Transmission of non-heated (NH) and heated (98C) RML, 22L and BSE isolates into *Tga20* mice.

Inoculum	Attack rate	ST (dpi ± SD)	ST increase (in days)	ST increase (%)	Statistical significance	WB profile^#^
RML NH	6/6	75 ± 7	67 days	89%	**	21 K
RML 98 °C	5/5	142 ± 26				
22L NH	7/7	112 ± 7	62 days	55%	**	21 K
22L 98 °C	6/6	174 ± 9				
BSE NH	6/6	164 ± 3	12 days	7%	None	19 K
BSE 98 °C	6/6	176 ± 21				

ST: Survival time, expressed as days post inoculation (dpi) ± standard deviation (SD).

Statistical significance was assessed by Mann-Whitney’s t test (*p-value < 0.05; **p-value < 0.01; ***p-value < 0.001).

^#^WB profile describes the size of the non-glycosilated band of the PrP^res^ protein which generally varies from 21 to 19 kD (21K or 19K) between different prion strains.

**Figure 3:**
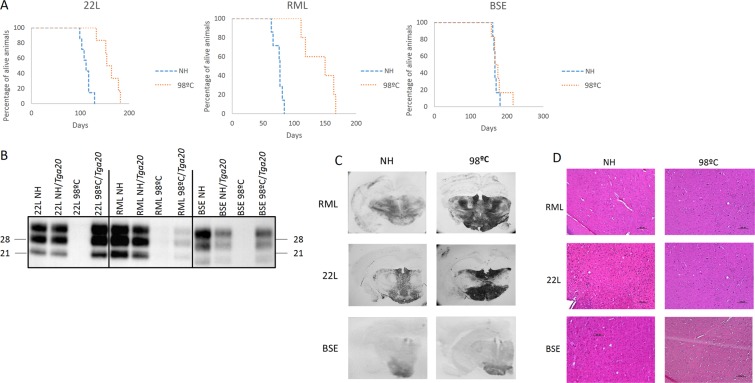
Transmission of non-heated (NH) and heated (98 °C) samples into *Tga20* mice. (**A**) Survival curves visually representing the data found in Table 1. (**B**) PrP^res^ levels of original heated and non-heated samples in comparison to brain PrP^res^ from *Tga20* mice challenged with heated and non-heated samples were detected in western blot with Sha31 mAb. Molecular weights in kD are shown. (**C**) Histological characterization of *Tga20* transgenic mice inoculated with RML, 22L and BSE heated and non-heated samples. Distribution of PrP^res^ deposits as assayed by PET-blot with Sha31 mAb showed no change between animals inoculated with heated or non-heated samples. (**D**) Histological characterization of *Tga20* transgenic mice inoculated with RML, 22L and BSE heated and non-heated samples. Hematoxylin and eosin showed no change between animals inoculated with heated or non-heated samples.

Survival curves visually representing this data can be found in Fig. 3A. Brain PrP^res^ of the inoculated animals proved to maintain the original electrophoretic signature of the prion strains (Fig. 3B). In the same line of results, for histopathological analysis no major differences of brain PrP^Sc^ distribution pattern was detected between animals infected with either heated or non-heated control samples (Fig. 3C,D). However increased intensity in the signal of the PET-blot pictures for animals inoculated with heated RML and 22L were detected, probably due to the longer survival time observed in these animals which could favor a greater accumulation of PrP^res^. Overall, this result indicates that no significant change in the neurological targeting of the three strains had occurred after heating.

### Differential effect of heat treatment in prion infectivity of different prion strains by mouse bioassay

The increases in ST observed in RML and 22L strains could have been the result of i) a decrease in prion infectivity, or ii) the appearance of new prion properties associated to PrP^Sc^ conformational alterations and therefore the emergence of a new different prion strain with longer ST. This last option was not much probable since previous results showed a concordance in both the WB profile and histological features. To fully discriminate between these two possibilities, heated and non-heated isolates were titrated by mouse bioassay. Ten-fold serial dilutions of these samples were intracranially inoculated in *Tga20* mice and the infectivity of each dilution assessed as a function of their mean ST (Table 2).

**Table 2:** Titration of non-heated (NH) and heated (98 °C) RML, 22L and BSE by end-point dilution in *Tga20* mice.

Inoculum	Inoculum dilution	Equivalent grams of inoculated brain	NH inocula		98° C inocula		Infectivity loss by mouse bioassay
			ST ± SD (n/n0)	Infectious titer (ID50/g)	ST ± SD (n/n0)	Infectious titer (ID50/g)	
RML	None	2 × 10^−3^	75 ± 7 (7/7)	1.58 × 10^7^	142 ± 23 (5/5)	3.79 × 10^1^	≈6 log_10_
	10^−1^	2 × 10^−4^	ND		ND		
	10^−2^	2 × 10^−5^	ND		151 ± 17 (3/7)		
	10^−3^	2 × 10^−6^	103 ± 22 (5/5)		>290 (0/4)		
	10^−4^	2 × 10^−7^	109 ± 17 (7/7)		>290 (0/4)		
	10^−5^	2 × 10^−8^	>174 (0/5)		ND		
	10^−6^	2 × 10^−9^	>174 (0/6)		ND		
22L	None	2 × 10^−3^	112 ± 13 (4/4)	1.58 × 10^7^	176 ± 33 (6/6)	1.58 × 10^2^	≈5 log_10_
	10^−1^	2 × 10^−4^	ND		ND		
	10^−2^	2 × 10^−5^	ND		188 ± 39 (4/4)		
	10^−3^	2 × 10^−6^	131 ± 10 (6/6)		>360 (0/6)		
	10^−4^	2 × 10^−7^	131 ± 21 (5/5)		>360 (0/7)		
	10^−5^	2 × 10^−8^	>230 (0/6)		>360 (0/6)		
	10^−6^	2 × 10^−9^	>230 (0/6)		ND		
BSE	None	2 × 10^−3^	154 ± 21 (5/5)	1.17 × 10^6^	176 ± 33 (5/5)	9.08 × 10^5^	≈0 log_10_
	10^−1^	2 × 10^−4^	ND		207 ± 31 (5/5)		
	10^−2^	2 × 10^−5^	179 ± 23 (5/5)		296 ± 81 (5/7)		
	10^−3^	2 × 10^−6^	>206 (1/4)		>392 (1/3)		
	10^−4^	2 × 10^−7^	>420 (0/4)		>364 (1/3)		
	10^−5^	2 × 10^−8^	>403 (0/6)		ND		
	10^−6^	2 × 10^−9^	ND		ND		

ND: Not done.

ST ± SD: Survival time ± standard deviation.

n/n_0_: Attack rate determined as the proportion of mice scored positive for brain PrP^res^ (n) from all the inoculated mice (n_0_).

As expected, no major differences in prion infectivity of heated and non-heated BSE samples were observed for any of the dilutions (Table 2), pointing to the high resistance to heat of this prion strain. By contrast, heat treatment decreased the infectious titer of RML and 22L prions by 6 and 5 log_10_, respectively.

### Differential effect of the heat treatment plus PK digestion on the prion templating activity of prion strains *in vitro*

**Figure 4:**
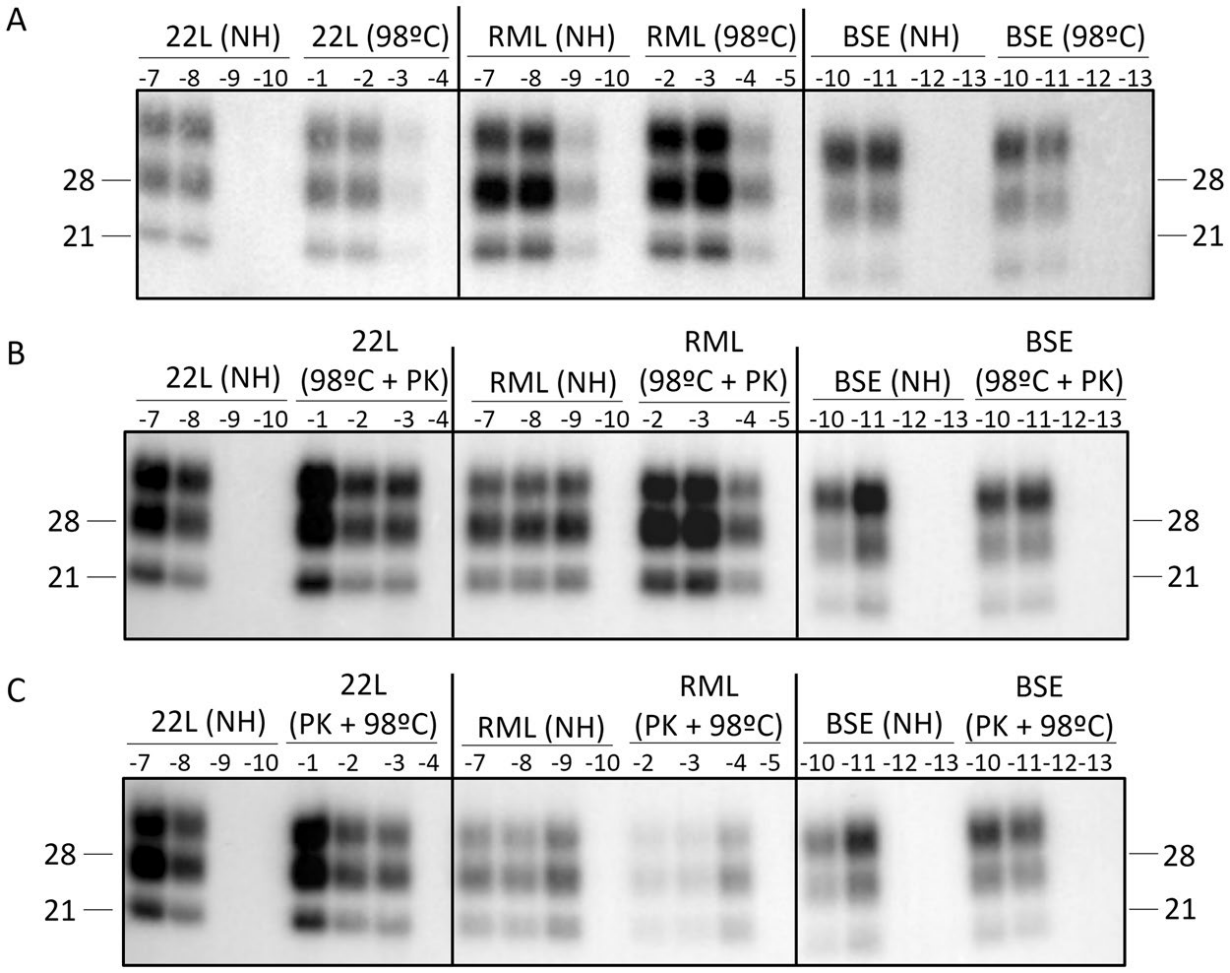
Templating activity analysis. Comparison of templating activity of non-heated (NH) with heated (98 °C) (**A**), heated and then PK digested (98 °C + PK) (**B**), PK digested and then heated (PK + 98 °C) (**C**) samples by PMCA on *Tga20* mouse brains analyzed by WB using the Sha31 mAb. 22L and RML treated samples showed a lower templating activity when compared to its non-heated counterparts.

The key event causing prion diseases, the PrP^C^ conversion into PrP^Sc^, has been successfully reproduced in cell-free conditions by PMCA^21^. This technique is able to amplify different prion strains in healthy brain homogenates from different species, maintaining their strain-specific biological, biochemical and infectious properties^22^. The higher sensitivity of the technique compared to bioassay makes it possible to test prion templating activity, meaning the capacity of the prion agent to sustain effective PrP^Sc^ amplification in serial dilutions as other way to perform prion titration detecting subinfectious levels of prions^23^. We thus used it as an alternative and more rapid approach for assessing the differential thermostability of prion strains. For that purpose, serial 10-fold dilutions of heated, heated plus PK digested, PK digested plus heated and untreated isolates were subjected to PMCA amplification using non-infected *Tga20* brain homogenate as substrate, as previously described^23^. The amplified material was then analyzed for PrP^res^ content by immunobloting. Conversion capacity of each strain was assessed and the loss of templating activity after the different treatments was compared to that obtained by mouse bioassay (Table 2). Non-heated RML and 22L prions exhibited high conversion capacities, resulting in positive amplification with brain material diluted up to 10^−9^ and 10^−8^–fold, respectively (Table 3). After heat treatment, RML and 22L amplified up to 10^−4^ and 10^−3^ dilutions, which indicate a templating activity loss of 5 log_10_ for RML and 22L (Table 3, Fig. 4). Exactly the same results were obtained when heat treatment was combined with PK digestion, regardless if the PK digestion was done before or after the heat pulse. By contrast BSE strain showed identical conversion capacities up to 10^−11^ dilutions both without and with the different treatments (Table 3, Fig. 4). The templating activity losses assessed for each strain mostly overlapped those infectivity losses previously determined by mouse bioassay of the plain heated and unheated samples which supports that PMCA would be a suitable and fast procedure for assessing thermostability of prion strains, instead of using an *in vivo* approach which consume more time and requires a higher use of animals.

**Table 3:** Conversion capacity of RML, 22L and BSE isolates before (NH) and after (98 °C) being heated at 98 °C for 2 hours.

Strain	Amplification NH	Amplification 98 °C	Amplification 98 °C + PK	Amplification PK + 98 °C	Templating activity loss by PMCA	Infectivity loss by mouse bioassay
22L	10^−8^	10^−3^	10^−3^	10^−3^	5 log_10_	≈5 log_10_
RML	10^−9^	10^−4^	10^−4^	10^−4^	5 log_10_	≈6 log_10_
BSE	10^−11^	10^−11^	10^−11^	10^−11^	0 log_10_	≈0 log_10_

## Discussion

Post-translational conversion of PrP^C^ into PrP^Sc^ causes a deep change in both protein structure and physicochemical features. In this study, three well defined mouse-adapted prion strains (BSE, RML and 22L), were treated with different conditions that involved heating at 98 °C for two hours combined or not with PK digestion and their biological and biochemical properties, as well as residual infectivity and seeding activity were assessed in comparison to untreated control strains.

Heat treatment caused an increase in the ST of inoculated mice when compared to mice challenged with unheated control material. Such increase was dependent on the prion strain being BSE the most thermostable strain (just a 7% increase in the mean ST) whereas RML and 22L were much more thermolabile (89% and 55% increase in the mean ST respectively). Increase in ST could be attributed to a shift of the prion strain as a consequence of the heat treatment. In a previous report studying prion thermostability, scrapie prion strain ME7 retained its general strain features while showing incubation periods longer than the controls at equivalent doses once autoclaved at 126 °C^24^. This result was interpreted as differences in the structure of the causal agent. However, none of the strain features like PrP^res^ glycoprofile, PrP^res^ deposition pattern and lesion profiles in the brains of inoculated mice were altered when comparing heated and non-heated isolates in our study. Therefore, it seems that the heat treatment just caused a reduction in the infectivity titer for our tested prion strains. Our end-point titration studies by both mouse bioassay and PMCA clearly proved that hypothesis finally discarding the possibility of a strain change. Indeed, PMCA also proved that combination of PK digestion with the heat treatment (either after or before the heat pulse) resulted in no change in the templating activity of the samples compared to plain heated samples. This work proves that PMCA can be used as a tool to discriminate between more or less thermostable strains faster than mouse bioassay.

It is important to note that heated isolates tested on this study showed differential reduction on infectivity (as proved by both mouse bioassay and PMCA) while showing high reductions in PrP^C^ and PrP^res^ by WB. The most extreme case is the one of BSE in which near total disappearance of PrP^res^ resulted in no reduction of the infectivity. Indeed, there are some kind of inverse relationship between the amount of PrP^res^ retained after heat treatment and maintenance of infectivity for the strains used on this study: 22L and RML retained some PrP^res^ but their infectivity was strongly affected whereas BSE infectivity was intact although showing no detectable PrP^res^. Partial and total dissociation between prion infectivity and PrP^res^ presence have been detected recurrently in the prion field. In the very beginning, such fact was used to claim against the Prion Protein-Only Hypothesis^25^. Later, attempts to clarify where exactly prion infectivity resides have been done although giving contradictory results. Regarding the size of the prion fibrils, for certain strains, infectivity was mapped to small and soluble PrP^Sc^ oligomers rather than larger fibrils^26,27^. Other studies, demonstrate the existence of protease-sensitive oligomers named sPrP^Sc^ (for “sensitive” PrP^Sc^) which may contribute to prion pathogenesis^28–31^ or not depending on the prion strain^32^. In this sense, our PMCA analysis using PK digested and heated material showed that the particle retaining the infectivity in the case of BSE is both resistant to heat and PK treatment. For 22L and RML, other PrP subpopulations affected for the different treatments subjected on this work were also responsible of part of the infectivity. At a subcellular level, strong correlation between infectivity and presence of lipid rafts have been traditionally done^33^. In fact, addition of certain lipids and polyanions to PMCA reactions tends to increase the efficiency of purified recombinant and cellular PrP conversion^34–36^. In contrast, other reports also point to presence of infectivity related to mitochondria and endoplasmic reticulum enriched fractions^37–39^. Thus, ours results provide another observation about dissociation of PrP^res^ and infectivity and more research is needed to clarify the puzzle conformed by PrP^res^, sPrP^Sc^ and other co-factors important for the maintenance of the infectivity. However, the possibility that each strain refold differently or require different refolding kinetic after the heat treatment could not be ruled out. The retained electrophoretic mobility and glycoform pattern argue against changes in the secondary and tertiary structure but quaternary structure could have been modified after the heat treatment, explaining the differences between strains.

Previous reports that studied differential resistance to heat in prion strains proposed the existence of a heteromeric structure involving two types of macromolecules in which one macromolecule, independent of the host, differs in its covalent structure between TSE strains^24,40^, being compatible with the “virino hypothesis” which proposes a host-independent informational molecule protected by the host protein PrP^41^. Later, the existence of prion strains as a result of different tertiary and quaternary structures while sharing the same amino acid sequence were proposed^42^. Therefore, both previous reports^24,37,43^ and our current results indicate that thermostability is a strain-specific physicochemical feature like the PrP^res^ glycoprofile, lesion profile or other well-known prion strain characteristics. Thus, it would be related to some extent to the PrP^Sc^ tertiary and/or quaternary structure. Regarding how prion molecules manage to survive to heat treatment, other authors postulated that dehydration of the prion agents by means of the removal of the water of solvation could stabilize the interactions between prion protein and some TSE agent-specific ligands^43^. In the same line, it was recently proposed that dehydration may also protect prion against inactivation via freezing and thawing processes^44^.

Two of the strains employed on this work, RML and 22L, are mouse scrapie isolates which proved to be very thermolabile. On the other hand, BSE was very much resistant to heat treatment. The ability to reach the brain in a faster way was linked to poor resistance to heat^18^. However, BSE is reported here as very stable being also well known for its high neuroinvasion capacity^45^. Indeed, BSE has already been described as an extremely stable prion strain and extremely resistant to environmental conditions^46–49^. As a matter of fact, BSE thermostability was related to its own origin, since spreading through meat-and-bone meal (MBM) implied heating of cow tissues for MBM production. Specifically, BSE emergence in the UK in the late 1980s is related to a replacement of batch dry-rendering plants by continuous-rendering systems which supposed that both the cooking temperature and the drying time were reduced^50^. Therefore, special efforts must be done to avoid residual prion contamination especially in the case of highly thermostable strains like BSE.

## Materials and Methods

### Ethics statement

Animal experiments were performed in strict accordance with the recommendations of the Code for Methods and Welfare Considerations in Behavioral Research with Animals (Directive 86/609EC and 2010/63/EU). Experiments in mice were carried out in CISA-INIA (Madrid) and authorized by the Committee on the Ethics of Animal Experiments (CEEA) of the Spanish Instituto Nacional de Investigación y Tecnología Agraria y Alimentaria (INIA); Permit Numbers: PROEX263/15 and CEEA2009/004.

### Prion samples

Three mouse adapted strains, BSE, RML and 22L, were used on this study. Such prion isolates have been obtained through serial transmission into mouse-PrP *Tga20* mice which overexpress the mouse wild-type PrP^C ^^51^. Prion isolates consisted on pooled brain homogenates (10% weight/volume in PBS).

### Heat treatment and PK digestion of prion strains

Several combinations of heat plus PK treatment were studied on this work: Plain heat treatment, heat treatment plus PK digestion and finally PK digestion plus heat treatment.

Heat treatment (98 °C): Brain homogenates of *Tga20* mice infected with BSE, RML or 22L strains were aliquoted (500 µl), placed in safe-lock tubes (Eppendorf) and heated at 98 °C for 2 hours in a thermocycler (Primus 96 Plus Thermal Cycler, MWG AG Biotech). Samples were removed, allowed to cool gradually to room temperature and harvested at −20 °C.

Heat treatment plus PK digestion (98 °C + PK): The heating process was done as stated above. After the gradually cooling of the samples, 250 µl were taken and aliquoted in 50 µl. Individual aliquots were mixed with 150 µl of healthy sheep brain and subjected to PK digestion with 40 µg/ml of PK using the reagents of the TeSeE (Bio-Rad) enzyme-linked immunosorbent assay at 37 °C for 15 min. The reaction was stopped by adding PMSF to a final concentration of 2 mM. Finally, samples were subjected to centrifugation (15,000 g, 7 min, 20 °C) and pellets were resuspended in 50 µl of PBS.

PK digestion plus heat treatment (PK + 98 °C): PK digestion was done as described above using as starting material 50 µl of unheated brain homogenates. After centrifugation, pellets were resuspended in 50 µl of PBS and subjected to heat treatment as described above.

### Total PrP and PrP^res^ detection by Western blot in the treated samples (inocula)

For total PrP detection, 15 µl of the different samples were analyzed by WB. Samples were prepared as previously detailed^52^. Serial dilutions were done by diluting the original sample into loading buffer just before electrophoresis. For immunoblotting, membranes were incubated with Sha31 mAb^19^ that recognizes 144-WEDRYYRE-151 epitope of the mouse-PrP sequence. To determine the presence of PrP^res^, 20 μl of the different samples were analyzed by WB as previously described^53^. For immunoblotting, membranes were incubated with Sha31 mAb. Immunocomplexes were detected with horseradish peroxidase-conjugated anti-mouse IgG (Amersham Pharmacia Biotech) after incubating the membranes for 1 hour. Immunoreactivity was visualized by chemiluminescence with ECL Select (GE Healthcare Amersham Biosciences). When necessary, WB images were quantified using ImageLab software v5.2.1(BioRad).

### Measurement of prion infectivity by mouse bioassay

The relative infectivity in the unheated and heated BSE, RML or 22L samples (10% brain homogenates) was calculated as a function of the survival times (ST) observed after their inoculation in *Tga20* mice. Briefly, groups of 6–9 individually identified (six to seven weeks old) *Tga20* mice were anesthetized with isoflurane and intracerebrally inoculated with 20 µl of the inocula (ten-fold serial dilution of each unheated and heated sample) in the right parietal lobe using a 25-gauge disposable hypodermic needle. Mice were examined biweekly for the development of neurological signs of the prion disease and humanely euthanized by cervical dislocation when progression of the disease was evident or at the end of the titration study: 2-fold the mean ST of the corresponding non-diluted sample for each strain. A mouse was considered positive for neurological disease when it showed two or three out of the 10 signs of neurological dysfunction previously described^54,55^. Once euthanized, brains were removed and analyzed for the presence of PrP^res^ by WB as described above. ST and attack rate were calculated for each inoculum. ST was expressed as the mean of the survival dpi of all the mice scored positive for brain PrP^res^, with its correspondent standard deviation (ST). Attack rate was determined as the proportion of mice scored positive for brain PrP^res^ from all the inoculated mice. Infectious dose (ID) was subsequently assessed by the Reed-Muench method^56^ and expressed in ID_50_ per gram (ID50 g^−1^) of tissue (Table 2) as previously described^57^.

### Statistical information

Mann-Whitney’s t test was applied to the ST data in order to find statistically significant differences between heated and un-heated inoculated animals. Significance level was adjusted to 0.05 and the test had two tails.

### PrP^res^ detection by Western blot in the brain of transgenic mice after mouse bioassay

A total of 175 mg of whole brain tissue was homogenized in 5% glucose in distilled water in grinding tubes (Bio-Rad) and adjusted to 10% (w/v) by using a TeSeE^TM^ Precess 48^TM^ homogenizer (Bio-Rad) following the manufacturer’s instructions. Homogenates were pressed through 0.4 mm needles of a calibration syringe and immediately frozen at −20 °C. To determine the presence of PrP^res^, 20 μl of 10% brain homogenates were analyzed by WB as described above. For immunoblotting, membranes were incubated with Sha31 mAb^19^.

### Measurement of prion templating activity by PMCA

Unheated and heated BSE, RML or 22L samples (10% brain homogenates) were titrated for their templating activity by miniaturized-bead PMCA (mb-PMCA)^23^. Mouse brain lysates from healthy *Tga20* mice were prepared and used as substrate. PMCA was performed in a final volume of 36 µl of lysate per well, in 96-well PCR microplate (Axygen, Union City, CA, USA). Each well was first filled with teflon beads (2.381 mm in diameter; Marteau et Lemarié, Pantin, France). A 4-µl aliquot of each original prediluted BSE, RML or 22L brain homogenates was suspended in 36 µl of healthy *Tga20* brain lysate to obtain the first 10^−3^ dilution. Then, a series of 10-fold dilutions were made. Microplates were placed on a Plexiglas rack designated for the cup horn of the Q700 sonicator (Misonix, Farmingdale, NY, USA, or Delta Labo, Colombelles, France) and subjected to 96 cycles of 30 s of sonication at 200–220 W power (~30% amplitude of the Q700 sonicator) followed by 29 min 30 s of incubation at 37 °C. The cup horn was filled with 300 ml of water circulating with rubber tubing in a water bath maintained at a temperature of 37 °C. At the end of the PMCA, the microplates were removed and aliquots from each sample were taken to be analyzed for their PrP^res^ content by dot- and WB analysis as previously described^23^. Negative controls were run in parallel. They were composed of unseeded substrate or seeded with uninfected brain homogenate.

## Supplementary information

Supplementary information file (full lenght blots)

## Data Availability

The authors declare that all other data supporting the findings of this study are available within the paper.
