# Supplementary material for: Thermostability as a highly dependent prion strain feature
Source: Sci Rep. 2019 Aug 6;9:11396. doi: 10.1038/s41598-019-47781-6 (PMC6684573; doi:10.1038/s41598-019-47781-6)

Supplementary information file
SREP-19-09295-T

Title: Thermostability as a highly dependent prion strain feature

Author list: Alba Marín-Moreno, Patricia Aguilar-Calvo, Mohammed Moudjou, Juan Carlos Espinosa, Vincent Béringue, Juan María Torres

Content: Full lenght blot of all figures

Figure 1

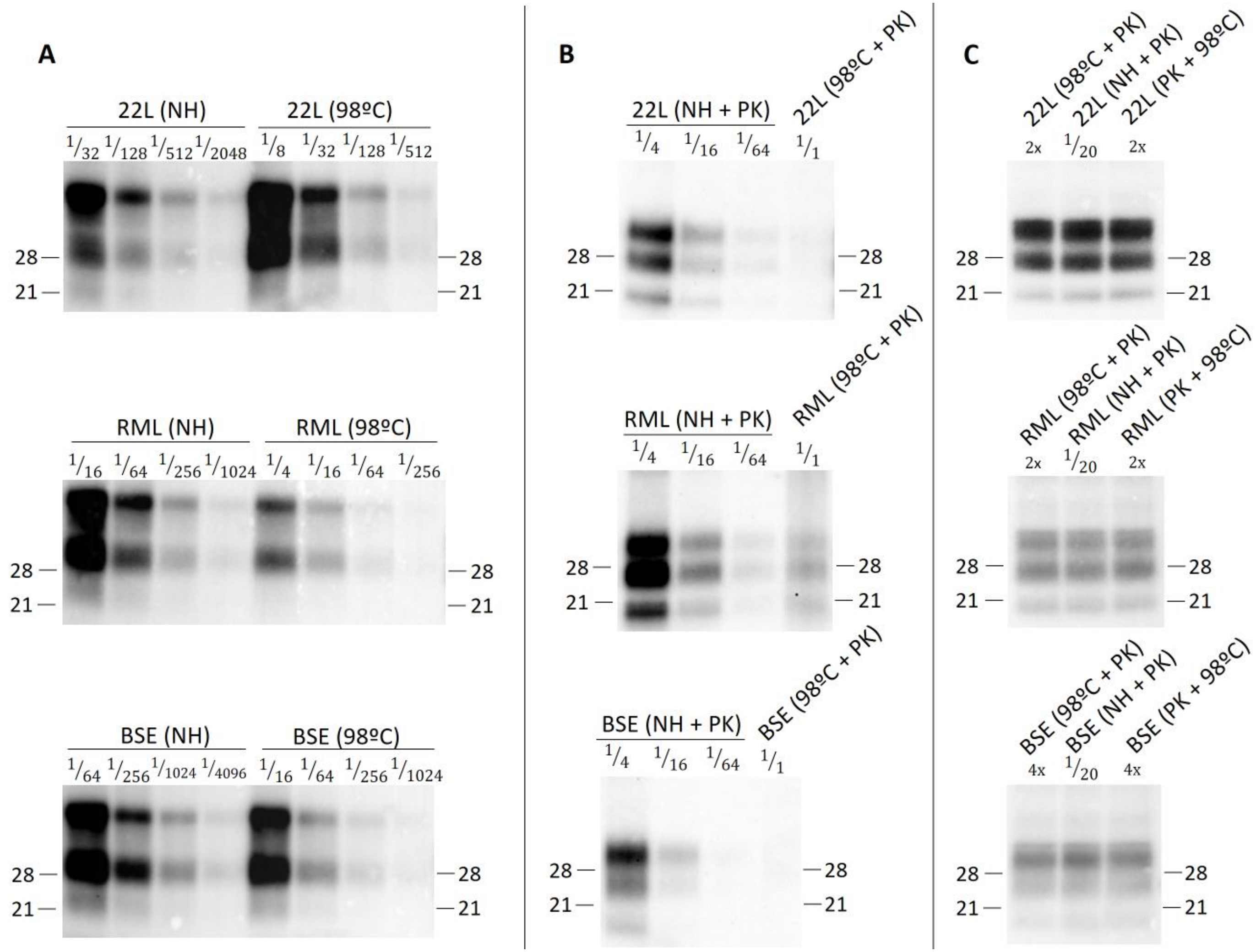

A
22L (NH)
22L (98ºC)
1/32 1/128 1/512 1/2048
1/8 1/32 1/128 1/512
28
21
RML (NH)
RML (98ºC)
1/16 1/64 1/256 1/1024
1/4 1/16 1/64 1/256
BSE (NH)
BSE (98ºC)
1/64 1/256 1/1024 1/4096
1/16 1/64 1/256 1/1024
B
22L (NH + PK)
22L (98ºC + PK)
1/4 1/16 1/64 1/1
RML (NH + PK)
RML (98ºC + PK)
BSE (NH + PK)
BSE (98ºC + PK)
C
22L (98ºC + PK)
22L (NH + PK)
22L (PK + 98ºC)
2x 1/20 2x
RML (98ºC + PK)
RML (NH + PK)
RML (PK + 98ºC)
BSE (98ºC + PK)
BSE (NH + PK)
BSE (PK + 98ºC)
4x 1/20 4x

Full lenght blots of figure 1

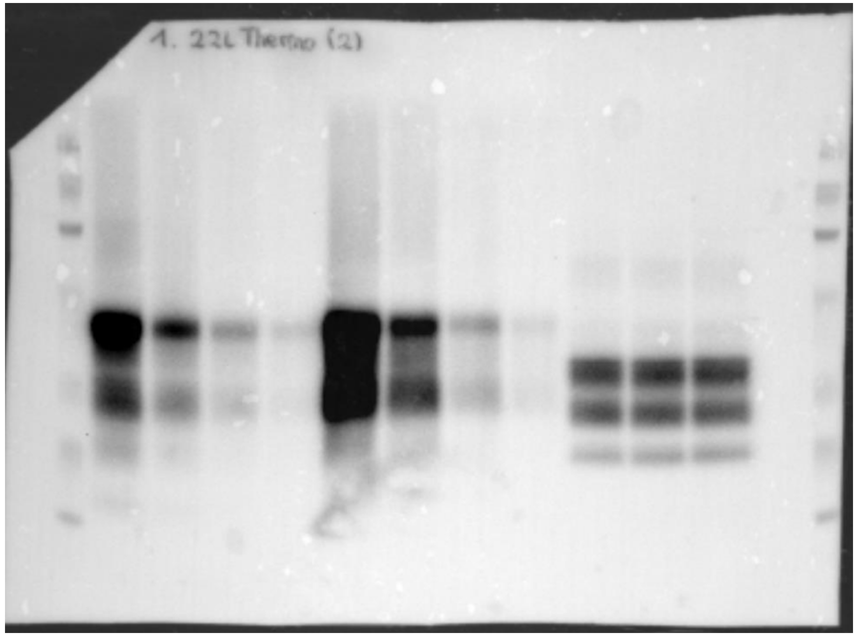

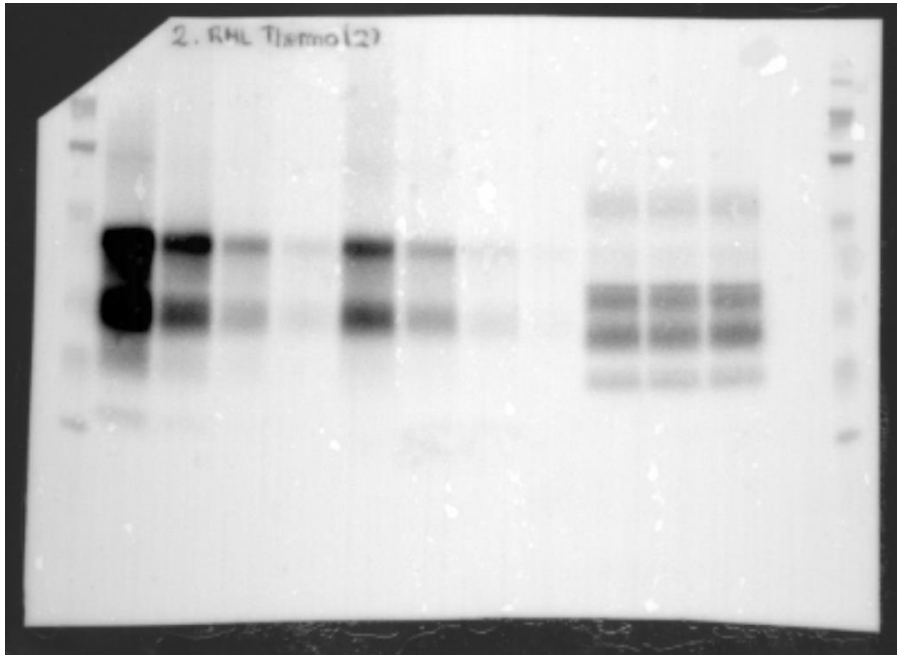

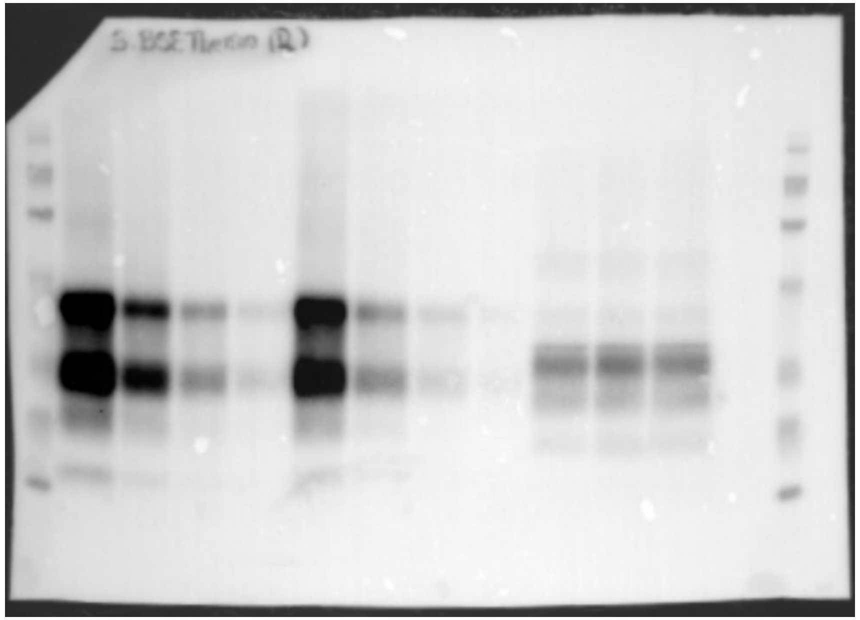

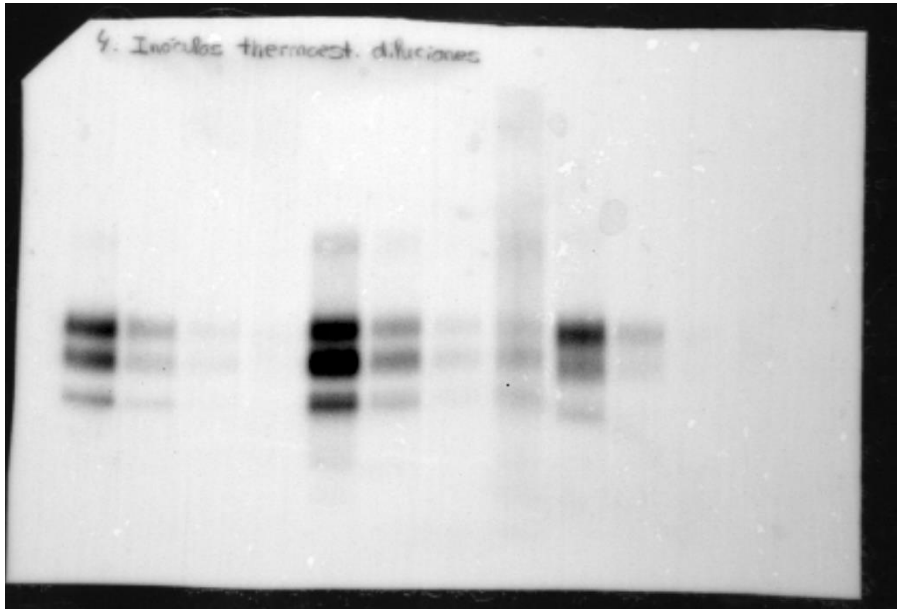

Figure 2

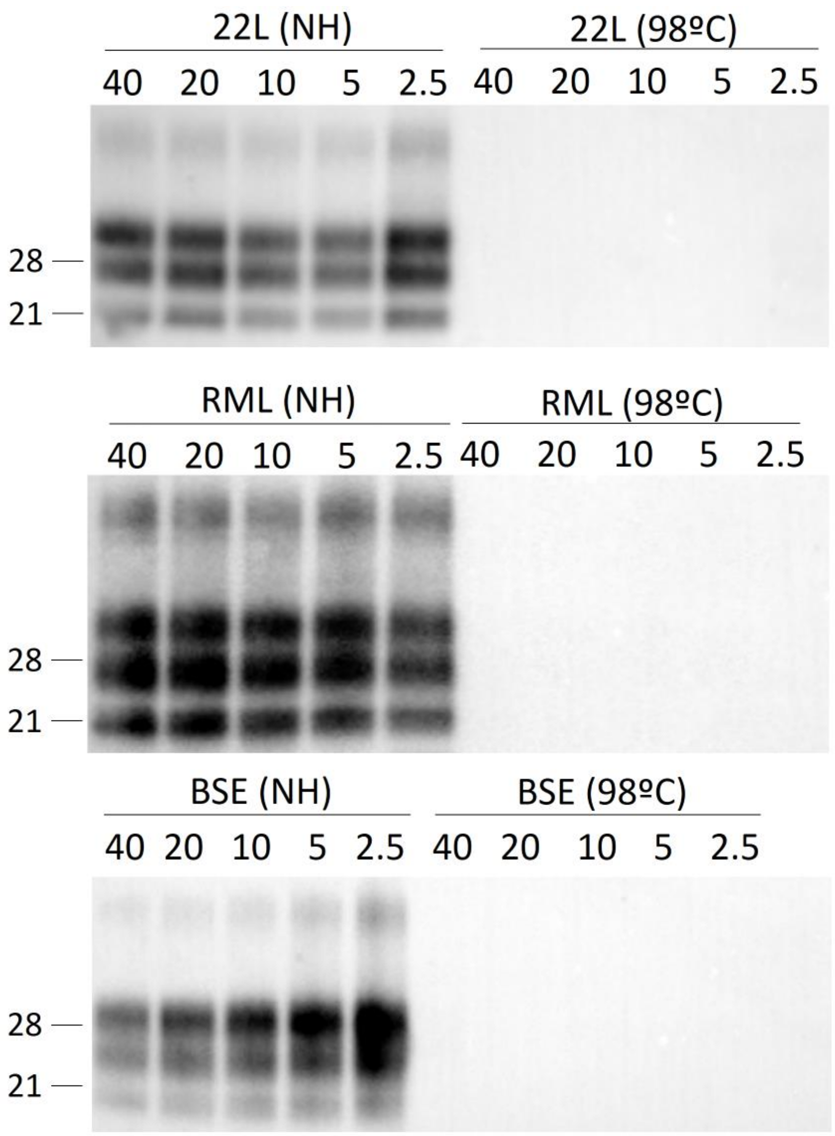

22L (NH)
22L (98ºC)
40 20 10 5 2.5 40 20 10 5 2.5
28
21
RML (NH)
RML (98ºC)
40 20 10 5 2.5 40 20 10 5 2.5
28
21
BSE (NH)
BSE (98ºC)
40 20 10 5 2.5 40 20 10 5 2.5
28
21

Full lenght blots of figure 2

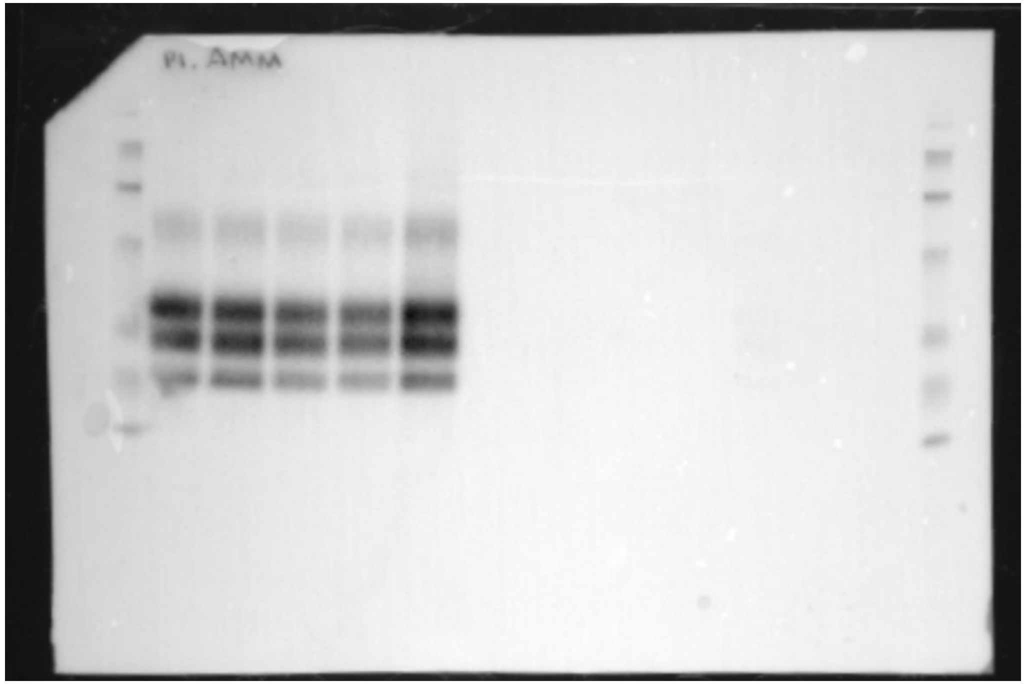

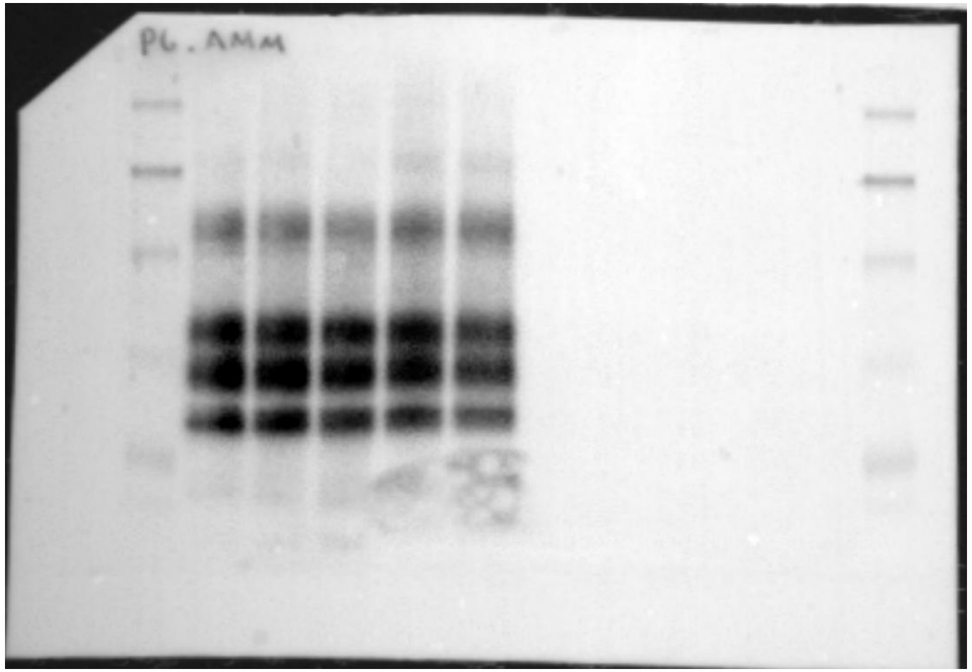

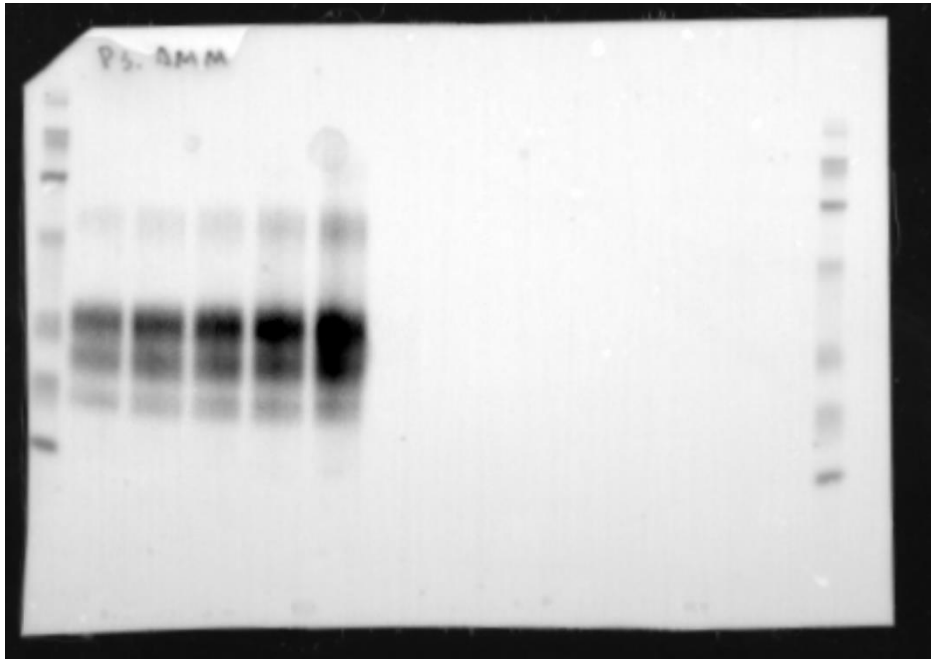

Figure 3

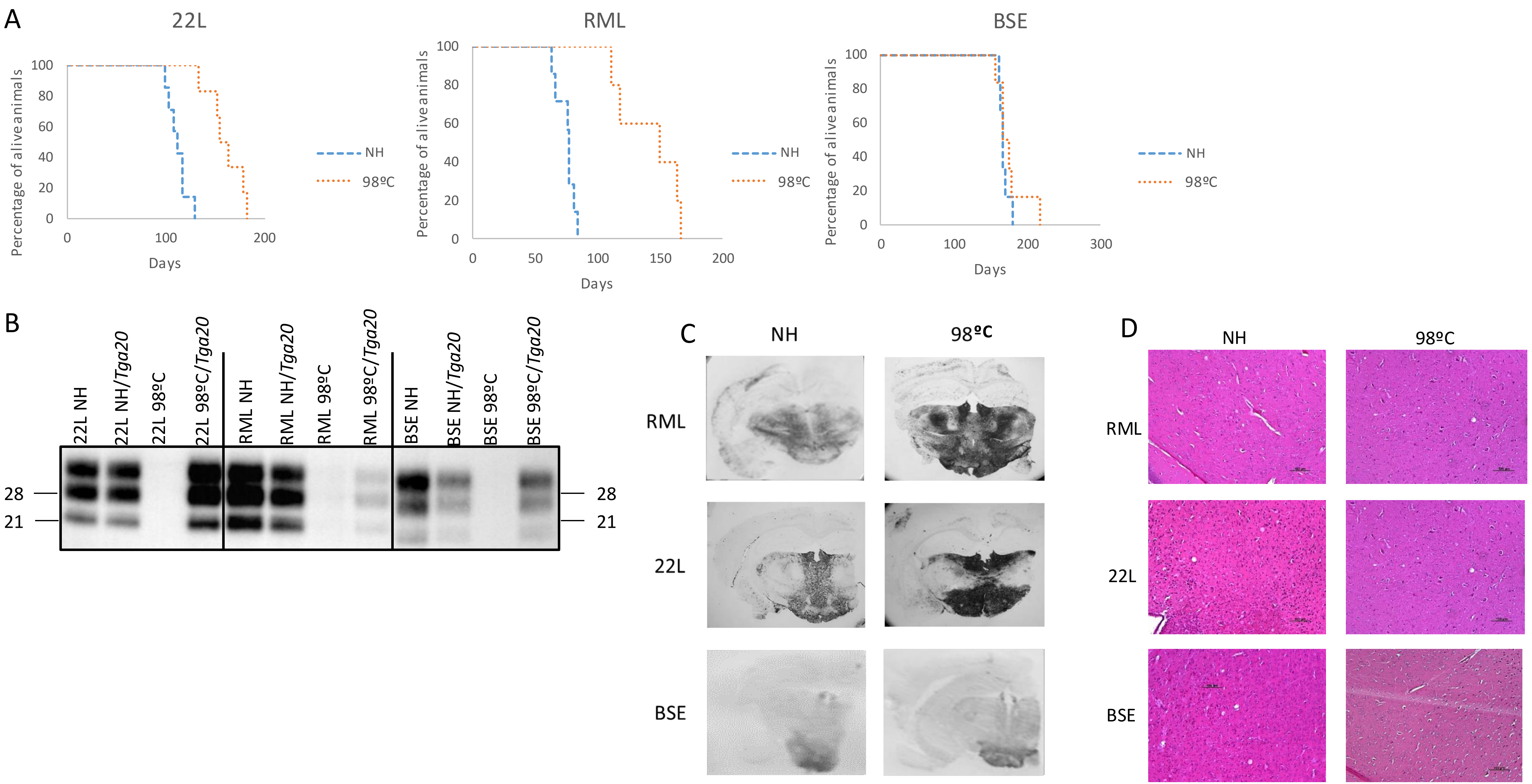

A
22L
RML
BSE
Percentage of alive animals
Days
NH
98ºC
B
22L NH
22L NH/Tga20
22L 98ºC
22L 98ºC/Tga20
RML NH
RML NH/Tga20
RML 98ºC
RML 98ºC/Tga20
BSE NH
BSE NH/Tga20
BSE 98ºC
BSE 98ºC/Tga20
28
21
C
NH
98ºC
RML
22L
BSE
D
NH
98ºC
RML
22L
BSE

Full lenght blot of figure 3

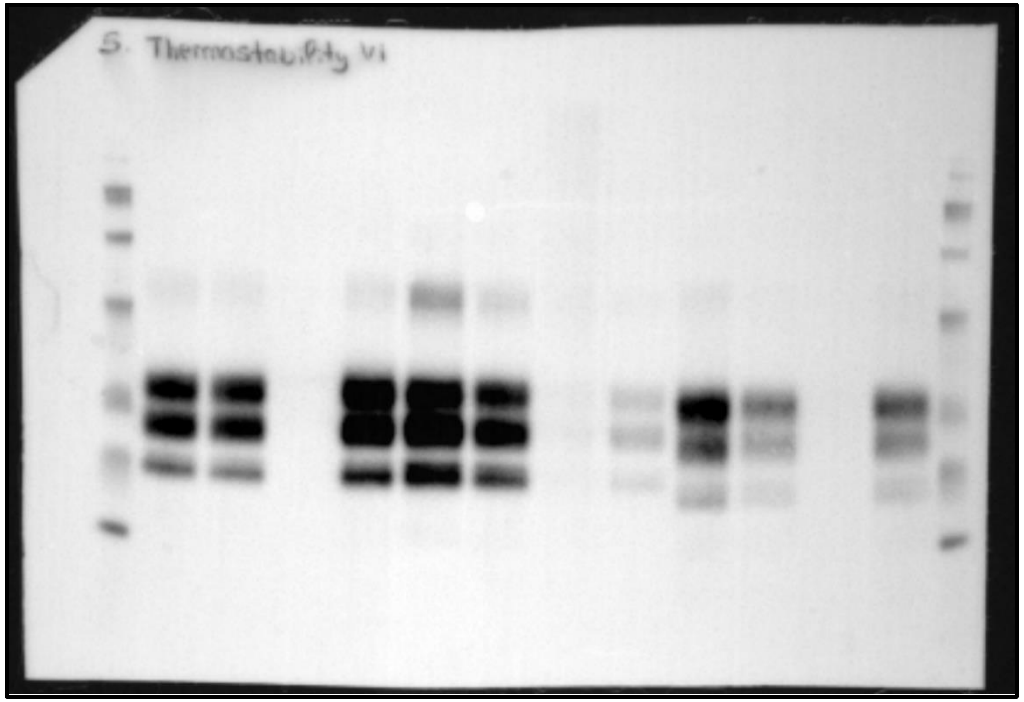

Figure 4

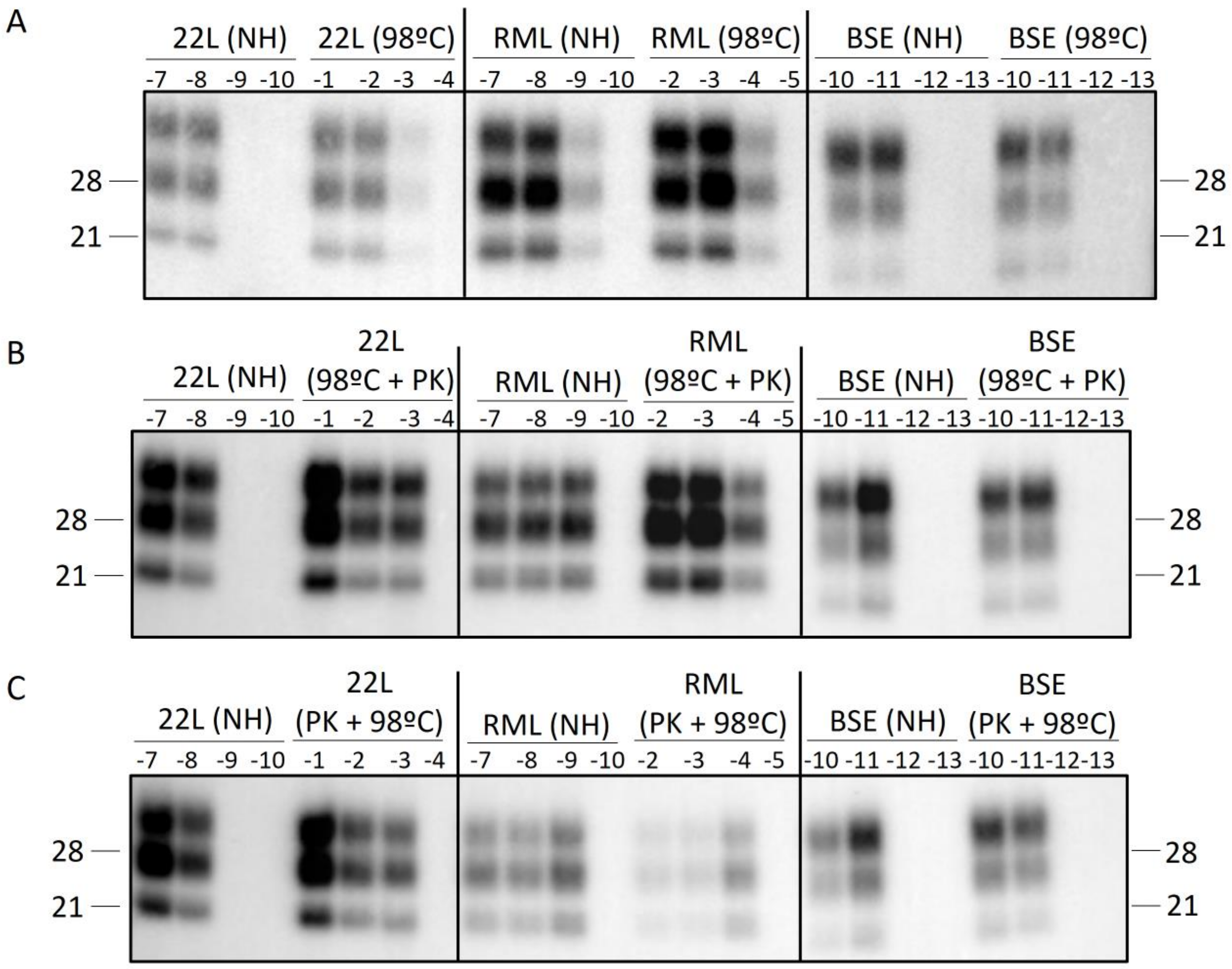

A
22L (NH)
22L (98ºC)
RML (NH)
RML (98ºC)
BSE (NH)
BSE (98ºC)
-7 -8 -9 -10 -1 -2 -3 -4 -7 -8 -9 -10 -2 -3 -4 -5 -10 -11 -12 -13 -10 -11 -12 -13
28
21
B
22L (NH)
22L (98ºC + PK)
RML (NH)
RML (98ºC + PK)
BSE (NH)
BSE (98ºC + PK)
-7 -8 -9 -10 -1 -2 -3 -4 -7 -8 -9 -10 -2 -3 -4 -5 -10 -11 -12 -13 -10 -11-12-13
28
21
C
22L (NH)
22L (PK + 98ºC)
RML (NH)
RML (PK + 98ºC)
BSE (NH)
BSE (PK + 98ºC)
-7 -8 -9 -10 -1 -2 -3 -4 -7 -8 -9 -10 -2 -3 -4 -5 -10 -11 -12 -13 -10 -11-12-13
28
21

Full lenght blots of figure 4

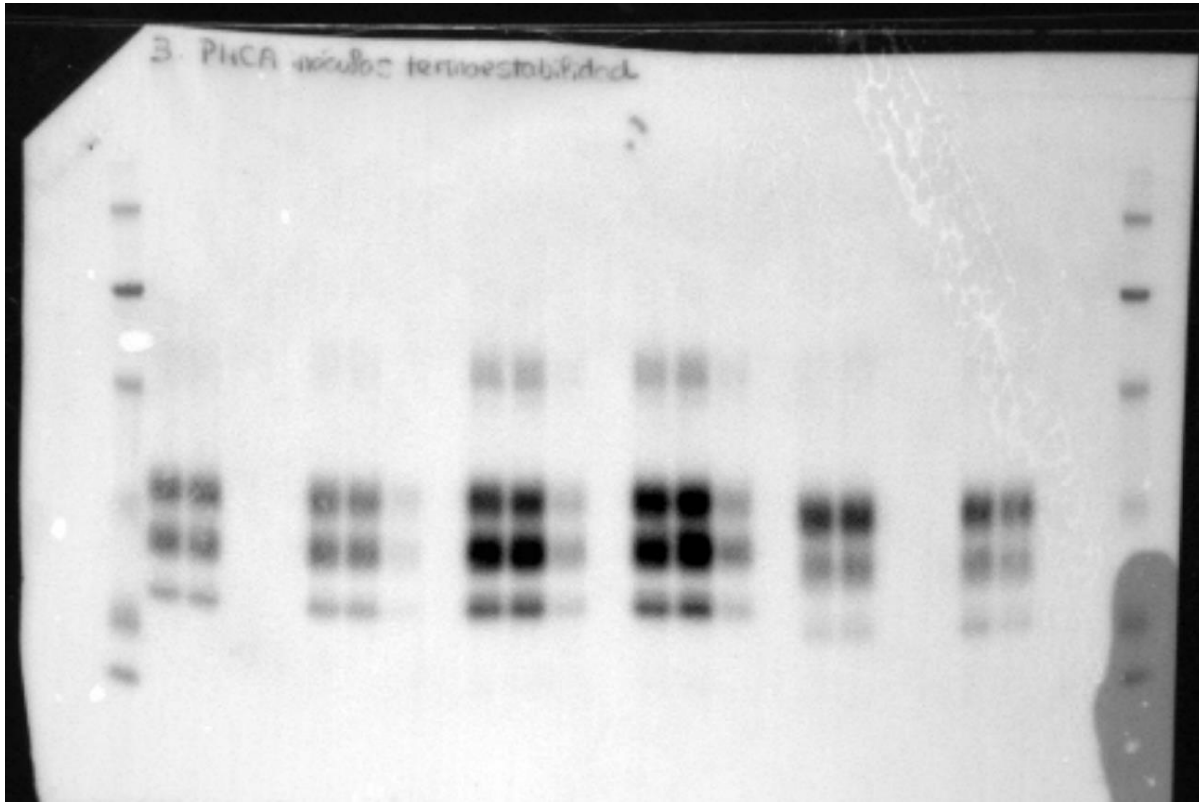

Full lenght blots of figure 4

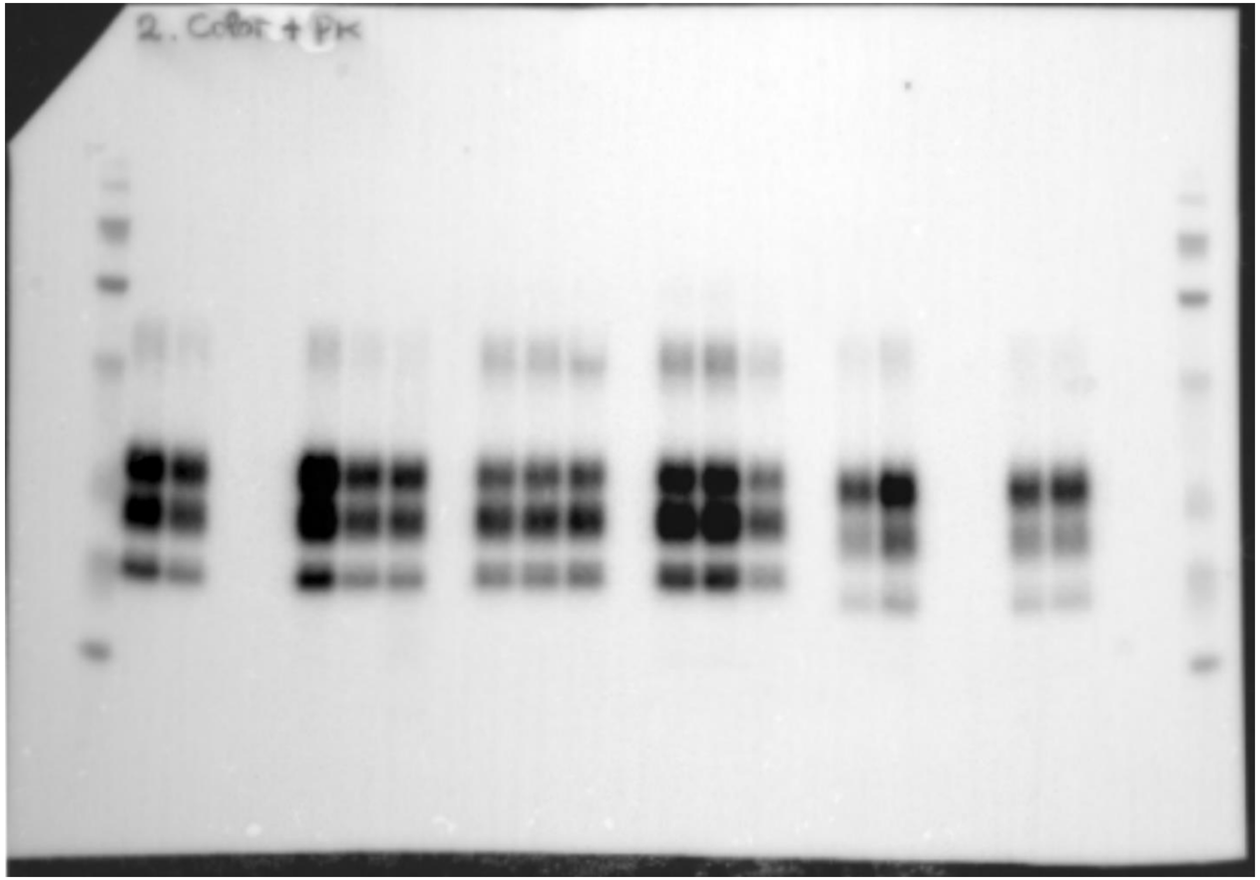

Full lenght blots of figure 4

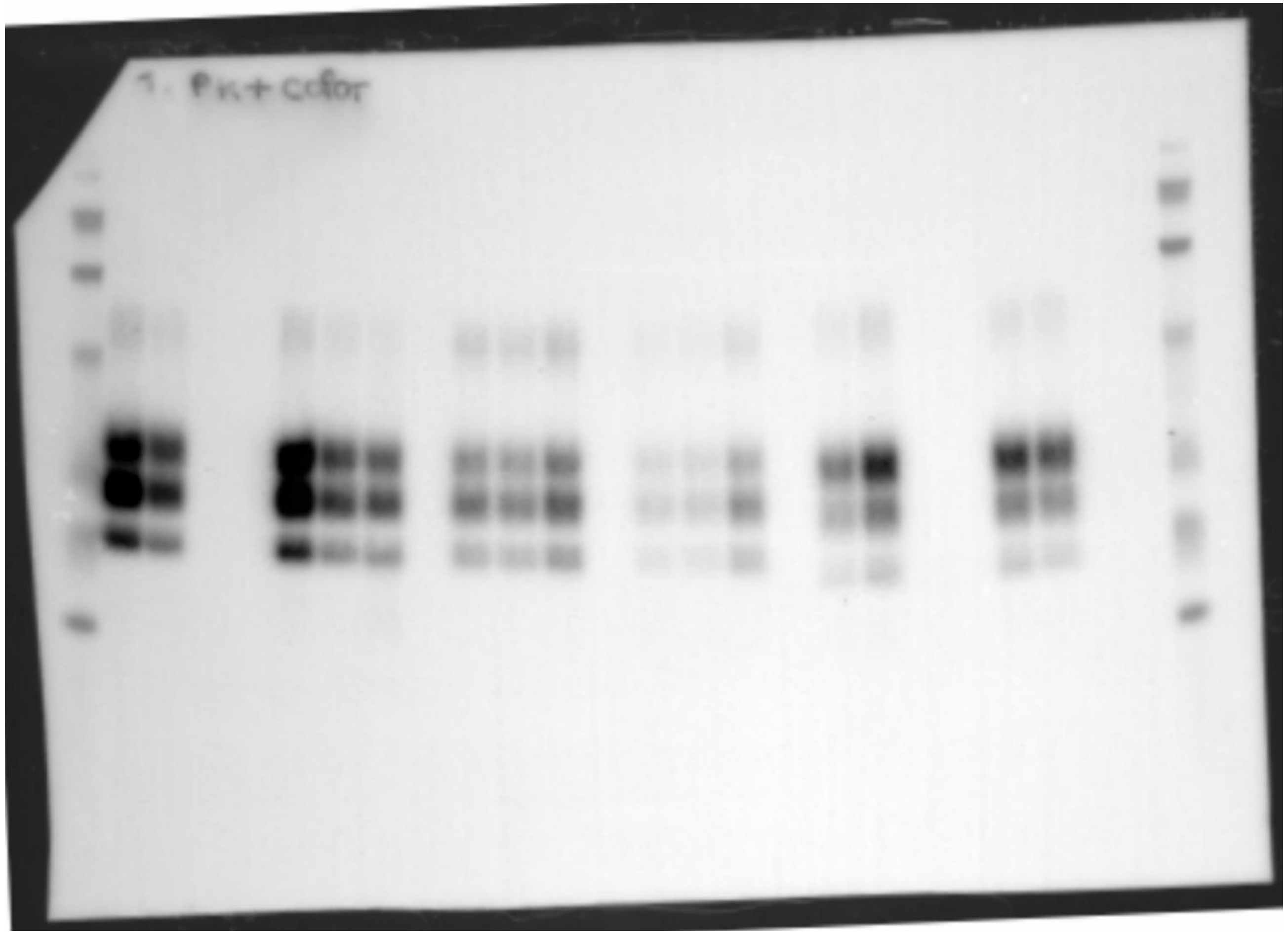

Supplement: Supplementary file 1 — Supplementary information file (full lenght blots) [file 41598_2019_47781_MOESM1_ESM.pdf]
